# Genetically linked brain imaging markers of memory decline in aging and Alzheimer's disease

**DOI:** 10.1002/alz.71663

**Published:** 2026-07-09

**Authors:** Yisu Yang, Anna Lorenz, Aditi Sathe, Kurt G. Schilling, Leslie S. Gaynor, Seo‐Eun Choi, Michael L. Lee, Phoebe Scollard, Emily H. Trittschuh, Shubhabrata Mukherjee, Jesse Mez, Logan C. Dumitrescu, Bennett A. Landman, Paul K. Crane, Michael L. Cuccaro, Timothy J. Hohman, Derek B. Archer

**Affiliations:** ^1^ Vanderbilt Memory and Alzheimer's Center Vanderbilt Health Nashville Tennessee USA; ^2^ Vanderbilt University Institute of Imaging Science Vanderbilt Health Nashville Tennessee USA; ^3^ Department of Medicine Vanderbilt Health Nashville Tennessee USA; ^4^ Division of Geriatric Medicine, Department of Medicine Vanderbilt Health Nashville Tennessee USA; ^5^ Department of Medicine University of Washington Seattle Washington USA; ^6^ Department of Psychiatry and Behavioral Sciences University of Washington School of Medicine Seattle Washington USA; ^7^ VA Puget Sound Health Care System, GRECC Seattle Washington USA; ^8^ Department of Neurology Boston University School of Medicine Boston Massachusetts USA; ^9^ Vanderbilt Genetics Institute Vanderbilt Health Nashville Tennessee USA; ^10^ Department of Biomedical Engineering Vanderbilt University Nashville Tennessee USA; ^11^ Department of Electrical and Computer Engineering Vanderbilt University Nashville Tennessee USA; ^12^ Department of Radiology & Radiological Sciences Vanderbilt Health Nashville Tennessee USA; ^13^ John P Hussman Institute for Human Genomics University of Miami Miller School of Medicine Miami Florida USA; ^14^ Department of Human Genetics Dr. John T. MacDonald Foundation, University of Miami Miami Florida USA

**Keywords:** aging, Alzheimer's disease, genetic covariance, memory, multimodal magnetic resonance imaging (MRI)

## Abstract

**INTRODUCTION:**

Memory is a strong endophenotype for Alzheimer's disease (AD) but is typically detectable only after substantial brain change. Genetically linking late‐life memory with mid‐life brain traits may identify early markers of AD‐related cognitive decline.

**METHODS:**

We leveraged GeNetic cOVariance Analyzer (GNOVA) to estimate genetic covariance between genome‐wide association studies (GWASs) of memory performance (MEM) and decline (memslopes) and 3935 UK Biobank (UKB) imaging‐derived phenotypes (IDPs) across diffusion, structural, and functional modalities, stratifying by cognitive status and apolipoprotein E (*APOE)* inclusion. Memory GWASs included 24,216 non‐Hispanic White older adults (mean = 74.46 years); IDP GWASs included 33,224 European‐ancestry mid‐life participants (45.1 to 81.8 years, mean = 64.28).

**RESULTS:**

Diffusion and structural IDPs in medial temporal and frontal regions showed the strongest genetic covariance with memory, with additional shared genetic architecture in default mode network functional connectivity.

**DISCUSSION:**

Mid‐life brain traits genetically linked to late‐life memory map to AD‐vulnerable regions, suggesting biologically relevant risk pathways and potential drug targets for cognitive decline.

## Highlights

- Diffusion and structural brain traits in the medial temporal lobe showed strongest genetic covariance with memory.
- Top functional brain traits highlighted connectivity in the default mode network.
- Shared genetic architecture between mid‐life brain imaging traits and late‐life memory parallels Alzheimer's disease vulnerability.

## 1 BACKGROUND

Alzheimer's disease (AD) is a progressive neurodegenerative disorder with hallmark pathologies of amyloid plaques and neurofibrillary tangles and underlies 60% to 80% of dementia cases.^1^, ^2^ Aside from efforts to characterize the genetic architecture of AD in the past decade via case/control genome‐wide association studies (GWASs),^3^, ^4^ recent work from our group using large‐scale harmonized data has shown that memory performance/decline is a highly heritable trait associated with AD.^5^, ^6^ Concurrently, large‐scale biometric datasets like the UK Biobank (UKB) have revealed that neuroimaging phenotypes, particularly structural and diffusion imaging measures, are also highly heritable.^7^, ^8^

However, due to a lack of paired genetic and neuroimaging data in large‐scale aging and AD cohorts, it is challenging to directly examine the genetic overlap between memory and brain structure and function with adequate power. Luckily, genetic correlation analyses between the UKB GWASs, currently the only large‐scale dataset linking genetics with comprehensive neuroimaging measures, and memory GWASs may provide an opportunity to capture these genetic relationships. Additionally, using mid‐life brain imaging data in the UKB allows us to identify robust and genetically relevant targets for AD‐related decline prior to overt brain atrophy, which may reveal risk pathways in early disease and inform future clinical trial design and therapeutic practices.

Here, we paired results from our previously published cognition‐stratified memory performance and decline GWASs in older adults^5^ with those from Smith et al.’s GWAS^8^ on 3935 brain imaging‐derived phenotypes (IDPs) of diffusion, structural, and functional modalities in the UKB to characterize shared genetic architecture between mid‐life brain structure and late‐life cognitive function in aging and AD. We hypothesized that IDPs showing the strongest genetic covariance with memory would highlight prefrontal, parietal, and default mode network (DMN) regions in the cognitively unimpaired analyses and medial temporal and posterior cingulate regions in the impaired analyses. We also expected to see the involvement of white matter tracts that our group previously demonstrated as being associated with cognitive performance, such as the fornix and cingulum.^9^, ^10^

## 2 METHODS

### 2.1 Summary statistics of memory performance and decline

Cross‐sectional memory performance (MEM) and longitudinal memory decline (memslopes) GWASs were obtained from our prior work,^5^ which leveraged harmonized memory performance measures^11^ from multiple longitudinal cohorts of cognitive aging and AD, including Adult Changes in Thought (ACT), Alzheimer's Disease Neuroimaging Initiative (ADNI), National Alzheimer's Coordinating Center (NACC), and Religious Orders Study/Rush Memory and Aging Project (ROS/MAP). In particular, the present study used summary statistics from GWASs of memory from self‐identified non‐Hispanic White (NHW) individuals with and without cognitive impairment. The impaired group included individuals with either mild cognitive impairment (MCI) or AD. Analyses were performed on memory performance (24,216 participants: 8465 impaired, 12,789 unimpaired) and memory decline (114,070 cognitive sessions; 19,707 participants: 6659 impaired, 11,264 unimpaired), with and without the apolipoprotein E (*APOE)* region. Participant characteristics for the memory GWASs by cohort are shown in Table S1. MEM (min = −3.02, max = 3.27, mean = 0.267) was quantified as a participant's harmonized memory score from the first cognitive visit; memslopes (min = −0.546, max = 0.244, mean = −0.078) was computed for participants with at least two cognitive visits. Specifically, memslopes was calculated by inputting harmonized memory scores into a null linear mixed‐effects regression model and letting the slope and intercept vary for each participant.

**RESEARCH IN CONTEXT**

1. **Systematic review**: We reviewed current literature using PubMed and Google Scholar to examine the individual and shared genetic architecture of memory and brain structure. Prior studies suggest high heritability for both memory performance/decline and neuroimaging measures of gray and white matter. However, there has been no comprehensive assessment of their genetic relationship due to the lack of paired genetic and neuroimaging data in large‐scale aging and Alzheimer's disease (AD) cohorts.
2. **Interpretation**: Our findings suggest that mid‐life brain imaging traits showing the strongest genetic covariance with late‐life memory parallel AD vulnerability in structure and function. These measures may reflect biologically relevant risk pathways and serve as potential drug targets for AD‐related cognitive decline.
3. **Future directions**: Our study demonstrates genetic pleiotropy between late‐life memory and mid‐life neuroimaging traits. Future work should aim to determine whether such relationships represent causal effects or linkage disequilibrium.

### 2.2 Summary statistics on UKB neuroimaging phenotypes of structural, diffusion, and resting‐state functional magnetic resonance imaging (MRI)

The second set of summary statistics was drawn from Smith et al.’s 2021 GWAS on brain‐imaging derived phenotypes (IDPs) in the UKB, conducted using neuroimaging data and genetic information from 33,224 individuals of European ancestry (45.1−81.8 years, mean = 64.28 years).^8^ This analysis expanded Elliot et al.’s 2018 GWAS on 3144 IDPs measured in 8428 individuals of the UKB to result in a total of 3935 IDPs spanning six modalities and quality control (QC) measures.^7^ In addition to the original QC measures (*n* = 16) in the UKB, the signal intensity and intensity contrast measures (*n* = 111) were also considered QC for our analyses. Of the non‐QC IDPs, 3802 were directly available from the UKB and consisted of 1325 T1‐weighted structural, one T2‐weighted fluid‐attenuated inversion recovery (T2‐FLAIR) structural, 14 susceptibility‐weighted MRI (SWI), 675 diffusion‐weighted MRI (dMRI), 16 task functional MRI (tfMRI), and 1771 resting‐state functional MRI (rfMRI) measures. The IDPs also included six summary functional connectivity features, which Smith et al. derived from individual resting‐state connectivity measures in the UKB. The list of all IDPs along with their modality and measure can be found in Table S2.

All IDPs were generated from an automated processing and QC pipeline that standardizes and quantifies raw MRI data.^12^ T1 images were non‐linearly warped to MNI152 space, then underwent FMRIB's Automated Segmentation Tool (FAST) and FMRIB's Integrated Registration and Segmentation Tool (FIRST) analyses for gray matter and subcortical segmentation, respectively.^13^, ^14^ Further FreeSurfer processing computed measures of cortical thickness, surface area, and volume for regions of interest (ROIs) based on several atlases, including the Desikan‐Killiany (Desikan) atlas, the Desikan‐Killiany‐Tourville (DKT) atlas, and the Destrieux atlas. dMRI data were first modeled with the diffusion tensor imaging fitting tool DTIFIT and NODDI (Neurite Orientation Dispersion and Density Imaging) to generate various white matter microstructural metrics, then underwent Tract‐Based Spatial Statistics (TBSS) and probabilistic tractography‐based analyses to generate white matter tracts. rfMRI time series data underwent group‐independent component analysis (ICA) at two spatial dimensions to generate node amplitude and edge connectivity measures with 25 and 100 components. Detailed information on the acquisition, processing, and QC of IDPs can be found in the UKB Brain Imaging Documentation (https://biobank.ctsu.ox.ac.uk/crystal/crystal/docs/brain_mri.pdf).

### 2.3 Resting‐state fMRI phenotype characterization

For all functional IDPs (i.e., node amplitude and edge connectivity measures), we created binary masks by setting a threshold for the absolute value of activation at z = 3.3, corresponding to a *p* value of 0.001. Then we applied the Schaefer 1000‐ROI parcellation^15^ to each IDP and calculated the percentage of its volume that overlapped with each of the seven Yeo resting‐state functional networks (visual, somatomotor, dorsal attention, ventral attention, limbic, frontoparietal, default mode).^16^ Calculations were done with FSL version 6.0.3. Full percent overlap results can be found in Table S3.

### 2.4 Statistical analysis

We performed munging of the UKB GWAS summary statistics using the linkage disequilibrium score regression (LDSC) method. Next, we leveraged the GeNetic cOVariance Analyzer (GNOVA)^17^ tool to estimate the genetic covariance of each UKB IDP with MEM and memslopes. GNOVA was conducted using European ancestry reference files from the 1000 Genomes project, which included a minor allele frequency cutoff of 5%. Analyses were stratified by cognitive status (all, impaired, unimpaired) and inclusion of the *APOE* region (chromosome 19, base pairs 43905796 to 45909395; with/without *APOE*), totaling 12 sets of GNOVA analyses for each IDP. Our main analyses were MEM and memslopes in all individuals, including the *APOE* region; remaining analyses were conducted as sensitivity analyses.

While GNOVA was conducted for all IDPs (*n* = 3935), we removed IDPs prior to subsequent analyses based on relevance and heritability. First, we removed all IDPs that were identified as a QC measure (*n* = 127). We also removed tfMRI IDPs (*n* = 16) and rfMRI node amplitude IDPs (*n* = 76) to highlight resting‐state measures and to avoid redundancy with rfMRI edge connectivity IDPs. Then we further grouped the IDPs into diffusion (dMRI), non‐diffusion structural (T1, T2‐FLAIR, SWI), and functional (rfMRI edge connectivity) modalities to calculate modality‐specific heritability cutoffs. We implemented a quantile approach to account for different distribution patterns across modalities, such that IDPs with heritability in the bottom quantile for each modality group were removed from subsequent analyses. Following this strategy, the heritability cutoff was 0.1688 for diffusion (506/675 post‐cutoff IDPs), 0.1244 for structural (1002/1336 post‐cutoff IDPs), and 0.0143 for functional (1276/1701 post‐cutoff IDPs). The heritability for all IDPs from the UKB GWASs and whether they passed the cutoff criteria can be found in Table S2.

Finally, we adjusted the *p* values for genetic covariance across all post‐cutoff IDPs within each set of GNOVA analyses (e.g., main MEM analysis with all individuals, including the *APOE* region) using the false discovery rate (FDR) method.^18^ All analyses were conducted in R versions 4.5.0 and 4.5.1, Python version 2.7.18, and Linux.

## 3 RESULTS

Results were stratified by outcome (MEM, memslopes), IDP modality (diffusion, non‐diffusion structural, functional), cognitive status (all, impaired, unimpaired), and inclusion of the *APOE* region (with/without *APOE*). The following sections focus on the main analysis with all individuals, including the *APOE* region. Table 1 provides the GNOVA statistics for top diffusion, non‐diffusion structural, and functional IDPs in the main MEM and memslopes analyses. Full cross‐sectional and longitudinal results for all 12 analyses can be found in Tables S4–S9, S10‐S15, respectively. Our results section highlights our most clinically significant findings.

**TABLE 1:** Top five structural, diffusion, and functional IDPs for all individuals with *APOE*.

MEM					
	IDP	ρ	SE_ρ_	*p_raw_*	*p_FDR_*
Diffusion	Mean MO in right fornix cres/stria terminalis	2.99×10^−2^	9.30×10^−3^	1.29×10^−3^	4.79×10^−2^
Structural	Area of right anterior circular sulcus of insula (Destrieux)	3.96×10^−2^	8.25×10^−3^	1.56×10^−6^	2.17×10^−3^
	Thickness of left lingual gyrus (DKT)	−3.76×10^−2^	8.85×10^−3^	2.16×10^−5^	7.52×10^−3^
	Thickness of left lingual gyrus (Desikan)	−3.75×10^−2^	8.83×10^−3^	2.18×10^−5^	7.52×10^−3^
	Volume of gray matter in left anterior left cingulate gyrus	3.73×10^−2^	8.82×10^−3^	2.31×10^−5^	7.52×10^−3^
	Volume of right putamen	4.17×10^−2^	9.88×10^−3^	2.43×10^−5^	7.52×10^−3^
Functional	Connectivity of edge 158 of 100‐dimensional ICA	3.92×10^−2^	7.96×10^−3^	8.50×10^−7^	2.17×10^−3^
	Connectivity of edge 101 of 100‐dimensional ICA	4.39×10^−2^	9.33×10^−3^	2.59×10^−6^	2.40×10^−3^
	Connectivity of edge 53 of 25‐dimensional ICA	3.74×10^−2^	8.35×10^−3^	7.49×10^−6^	5.21×10^−3^
	Connectivity of edge 12 of 100‐dimensional ICA	3.72×10^−2^	8.79×10^−3^	2.27×10^−5^	7.52×10^−3^
	Connectivity of edge 345 of 100‐dimensional ICA	−3.29×10^−2^	7.97×10^−3^	3.73×10^−5^	9.88×10^−3^

Memslopes					
	IDP	ρ	SE_ρ_	*p_raw_*	*p_FDR_*
Diffusion	Mean OD in right superior cerebellar peduncle	−3.84×10^−2^	1.05×10^−2^	2.72×10^−4^	1.76×10^−2^
	Mean MO in left superior corona radiata	−3.55×10^−2^	1.06×10^−2^	7.68×10^−4^	3.69×10^−2^
	Weighted‐mean L1 in left corticospinal tract	−5.97×10^−2^	1.81×10^−2^	9.84×10^−4^	4.03×10^−2^
Structural	Area of right pars orbitalis (DKT)	4.95×10^−2^	1.08×10^−2^	5.10×10^−6^	3.55×10^−3^
	Thickness of left superior and transverse occipital sulci (Destrieux)	−4.95×10^−2^	1.14×10^−2^	1.45×10^−5^	4.47×10^−3^
	Thickness of left medial occipitotemporal and lingual sulci (Destrieux)	−4.74×10^−2^	1.17×10^−2^	5.00×10^−5^	9.19×10^−3^
	Area of right pars orbitalis (Desikan)	4.27×10^−2^	1.07×10^−2^	6.46×10^−5^	9.19×10^−3^
	Thickness of left superior occipital gyrus (Destrieux)	−4.67×10^−2^	1.17×10^−2^	6.78×10^−5^	9.19×10^−3^
Functional	Connectivity of edge 1 of 100‐dimensional ICA	−5.20×10^−2^	9.12×10^−3^	1.16×10^−8^	3.23×10^−5^
	Connectivity of edge 118 of 25‐dimensional ICA	3.78×10^−2^	7.61×10^−3^	6.63×10^−7^	7.77×10^−4^
	Connectivity of edge 292 of 100‐dimensional ICA	4.01×10^−2^	8.14×10^−3^	8.38×10^−7^	7.77×10^−4^
	Connectivity of edge 121 of 25‐dimensional ICA	3.46×10^−2^	7.72×10^−3^	7.36×10^−6^	3.76×10^−3^
	Connectivity of edge 938 of 100‐dimensional ICA	3.75×10^−2^	8.39×10^−3^	8.11×10^−6^	3.76×10^−3^

Abbreviations: Desikan, Desikan‐Killiany atlas; Destrieux, Destrieux atlas; DKT, Desikan‐Killiany‐Tourville atlas; ICA, independent component analysis, MEM, cross‐sectional memory performance; memslopes, longitudinal memory decline; MO, tensor mode; OD, orientation dispersion index; *p*_FDR_, *p* value for genetic covariance estimate after adjusting for multiple comparisons; *p_raw_*, raw *p* value for genetic covariance estimate; SE_ρ_, standard error of genetic covariance estimate; ρ, genetic covariance estimate rho.

### 3.1 Genetic covariance between structural IDPs and cross‐sectional memory performance (MEM)

**FIGURE 1:**
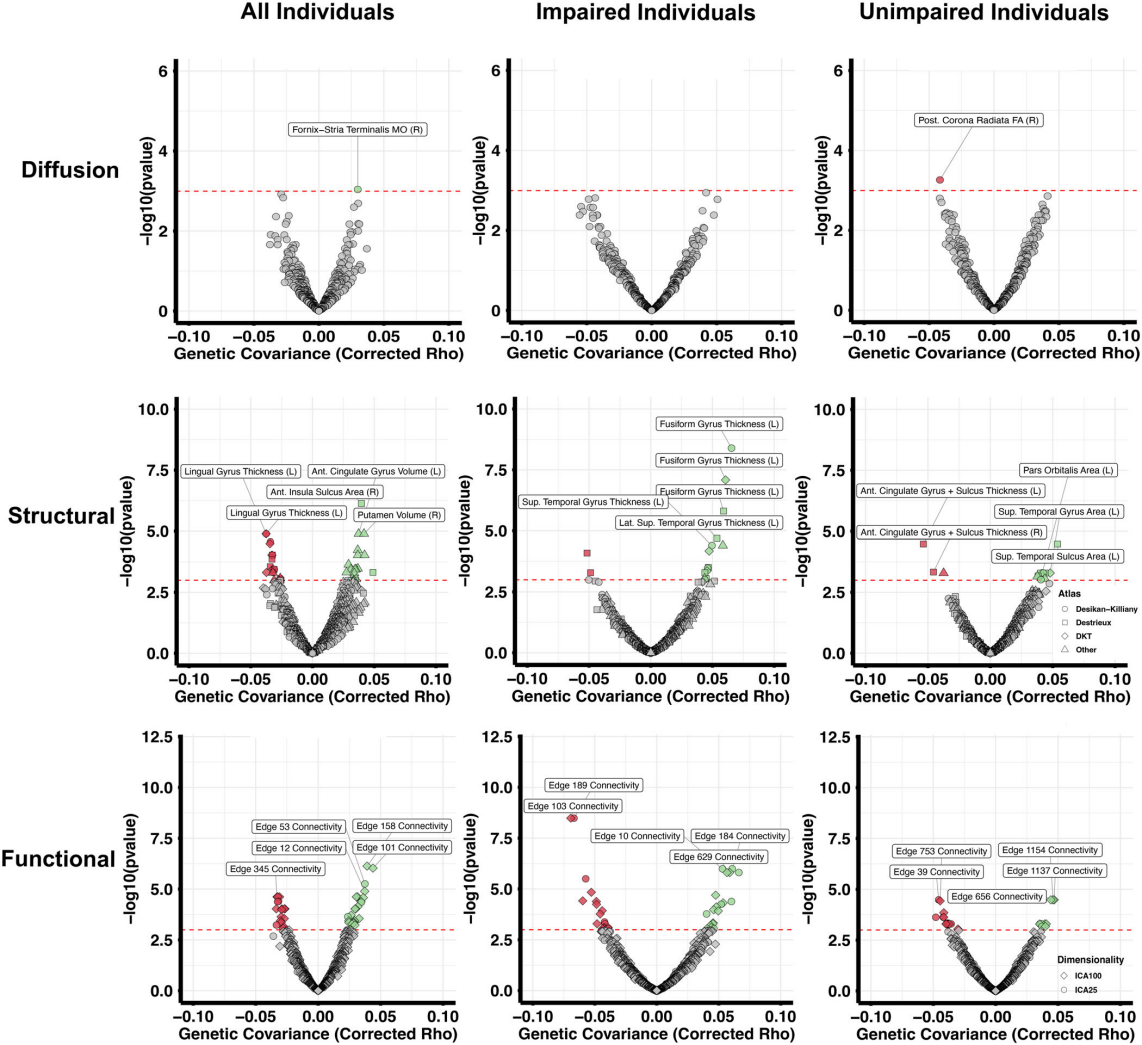
Genetic covariance between imaging‐derived phenotypes (IDPs) and memory performance. Volcano plots show strength of genetic covariance of diffusion (top panel), structural (middle panel), and functional (bottom panel) IDPs with cross‐sectional memory performance (MEM) for all, impaired, and unimpaired individuals, including the *APOE* region in the analyses. Colors highlight significant genetic covariance with memory (FDR‐corrected *p* < 0.05), with green indicating positive covariance and red indicating negative covariance. Data point shape for structural IDPs indicates the atlas used to generate the cortical measure, with circles representing the Desikan‐Killiany atlas, squares representing the Destrieux atlas, diamonds representing the Desikan‐Killiany‐Tourville (DKT) atlas, and triangles representing other smaller atlases (e.g., thalamic nuclei, hippocampal subfields). Data point shape for functional IDPs indicates the dimensionality of group ICA, with diamonds representing 100‐dimensional ICA and circles representing 25‐dimensional ICA. Top IDPs for each modality and analysis that survived FDR correction are labeled. Diffusion measures highlight regions such as the fornix and corona radiata; structural measures highlight regions such as the lingual gyrus, anterior cingulate gyrus and sulcus, fusiform gyrus, and superior temporal gyrus and sulcus. Table S18 provides the mapping between original UKB IDP names and our intuitive names. Ant., anterior; L, left hemisphere; Lat., lateral; Post., posterior; R, right hemisphere; Sup., superior.

Figure 1 (top and middle panels) illustrates the top diffusion and non‐diffusion structural IDPs that exhibited the greatest genetic covariance with MEM in all, impaired, and unimpaired individuals, including the *APOE* region. The top diffusion IDP in the main analysis was tensor mode (MO) of the right fornix cres‐stria terminalis (ρ = 0.030, SEρ = 9.30 × 10−3, *p*_FDR_ = 4.79 × 10−2, praw = 1.29 × 10−3). Sensitivity analyses identified the posterior corona radiata in unimpaired individuals. The top non‐diffusion structural IDPs in the main analysis included the area of the anterior segment of the circular sulcus of the right insula in the Destrieux atlas (ρ = 0.040, SE_ρ_ = 8.25 × 10^−3^, *p*_FDR_ **= 2.17 × 10^−3^, *p_raw _* = 1.56 × 10^−6^), thickness of the left lingual gyrus in both DKT and Desikan atlases (DKT: ρ = −0.038, SE_ρ_ = 8.85 × 10^−3^, *p*_FDR_ **= 7.52 × 10^−3^, *p_raw _* = 2.16 × 10^−5^; Desikan: ρ = −0.038, SE_ρ_ = 8.83 × 10^−3^, *p*_FDR_ **= 7.52 × 10^−3^, *p_raw _* = 2.18 × 10^−5^), gray matter volume of the anterior division of the left cingulate gyrus (ρ = 0.037, SE_ρ_ = 8.82 × 10^−3^, *p*_FDR_ **= 7.52 × 10^−3^, *p_raw _* = 2.31 × 10^−5^), and volume of the right putamen (ρ = 0.042, SE_ρ_ = 9.88 × 10^−3^, *p*_FDR_ **= 7.52 × 10^−3^, *p_raw _* = 2.43 × 10^−5^). Sensitivity analyses identified fusiform gyrus in impaired individuals, anterior cingulate gyrus and sulcus in unimpaired individuals, and superior temporal gyrus in both.

**FIGURE 2:**
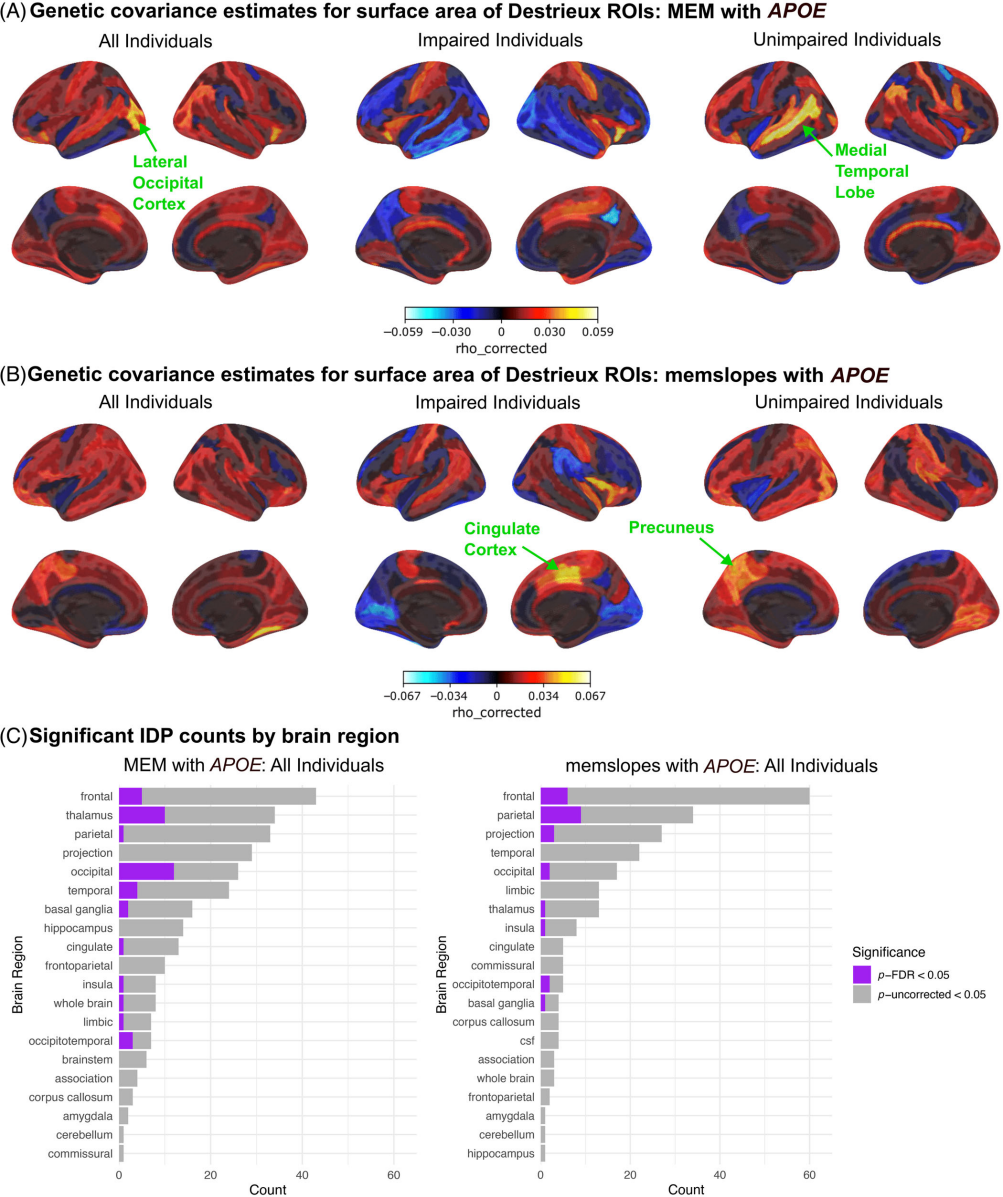
Summary of structural and diffusion imaging‐derived phenotype (IDP) covariance with memory. Destrieux surface area regions of interest (ROIs) are colored by genetic covariance with MEM (A) and memslopes (B) in all, impaired, and unimpaired individuals, including the *APOE* region. Warm colors indicate positive covariance, cool colors indicate negative covariance. Highlighted regions include the lateral occipital cortex, medial temporal lobe, posterior cingulate cortex, and precuneus. Bar charts illustrate the number of significant IDPs before and after FDR correction grouped by brain region (C), with most counts in frontal, parietal, and occipital regions as well as the thalamus.

Figure 2A illustrates surface area measures from the Destrieux atlas colored by genetic covariance with MEM in all, impaired, and unimpaired individuals, including the *APOE* region. Highlighted regions include the lateral occipital cortex and medial temporal lobe. The total number of significant structural IDPs for MEM grouped by brain region is illustrated in Figure 2C (left), with the highest counts in the frontal and occipital regions as well as the thalamus.

### 3.2 Genetic covariance between structural IDPs and longitudinal memory decline (memslopes)

**FIGURE 3:**
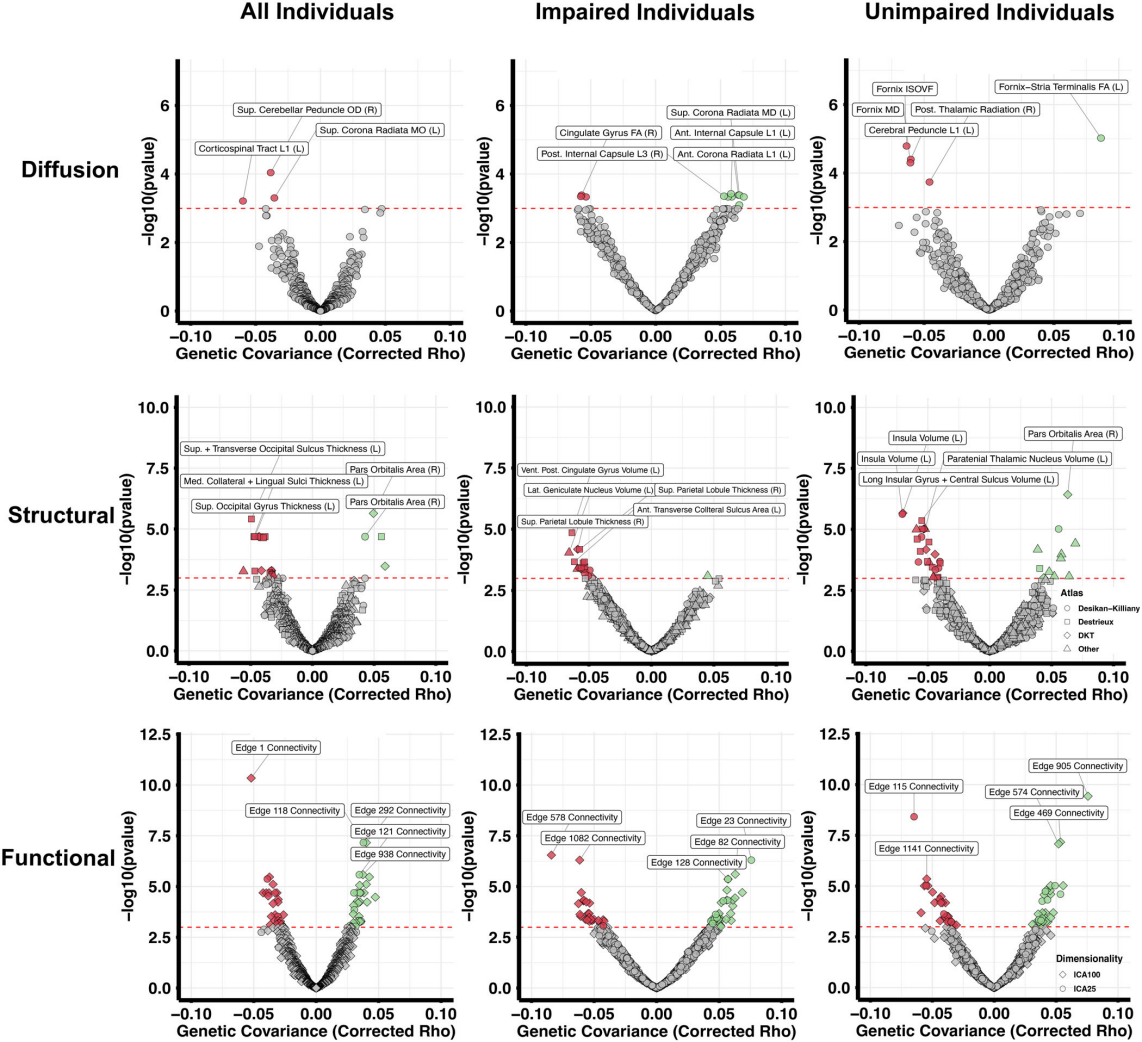
Genetic covariance between imaging‐derived phenotypes (IDPs) and memory decline. Volcano plots show strength of genetic covariance of diffusion (top panel), structural (middle panel), and functional (bottom panel) IDPs with longitudinal memory decline (memslopes) for all, impaired, and unimpaired individuals, including the *APOE* region in the analyses. Colors highlight significant genetic covariance with memory (FDR‐corrected *p* < 0.05), with green indicating positive covariance and red indicating negative covariance. Data point shape for structural IDPs indicates the atlas used to generate the cortical measure, with circles representing the Desikan‐Killiany atlas, squares representing the Destrieux atlas, diamonds representing the Desikan‐Killiany‐Tourville (DKT) atlas, and triangles representing other smaller atlases (e.g., thalamic nuclei, hippocampal subfields). Data point shape for functional IDPs indicates the dimensionality of group ICA, with diamonds representing 100‐dimensional ICA and circles representing 25‐dimensional ICA. Top IDPs for each modality and analysis that survived FDR correction are labeled. Diffusion measures highlight regions such as the fornix, cingulum, and posterior thalamic radiation; structural measures highlight regions such as the pars orbitalis, posterior cingulate gyrus, superior occipital gyrus and sulcus, and insula. Table S18 provides the mapping between the original UKB IDP names and our intuitive names. Ant., anterior; L, left hemisphere; Lat., lateral; Med., medial; Post., posterior; R, right hemisphere; Sup., superior; Vent., ventral.

Figure 3 (top and middle panels) illustrates the top diffusion and non‐diffusion structural IDPs that exhibited greatest genetic covariance with memslopes in all, impaired, and unimpaired individuals, including the *APOE* region. Top diffusion IDPs in the main analysis included the orientation dispersion (OD) index of the right superior cerebellar peduncle (ρ = −0.038, SE_ρ_ = 0.011, *p*_FDR_ **= 0.018, *p_raw _* = 2.72 × 10^−4^), MO of the left superior corona radiata (ρ = −0.036, SE_ρ_ = 0.011, *p*_FDR_ **= 0.037, *p_raw _* = 7.68 × 10^−4^), and L1 of the left corticospinal tract (ρ = −0.060, SE_ρ_ = 0.018, *p*_FDR_ **= 0.040, *p_raw _* = 9.84 × 10^−4^). Sensitivity analyses identified cingulate gyrus in impaired individuals and fornix and thalamic radiation in unimpaired individuals. Top non‐diffusion structural IDPs in the main analysis included the area of the right pars orbitalis in both DKT and Desikan atlases (DKT: ρ = 0.050, SE_ρ_ = 0.011, *p*_FDR_ **= 3.55 × 10^−3^, *p_raw _* = 5.10 × 10^−6^; Desikan: ρ = 0.043, SE_ρ_ = 0.011, *p*_FDR _= 9.19 × 10^−3^, *p_raw _* = 6.46 × 10^−5^), thickness of the left superior occipital sulcus and transverse occipital sulcus in the Destrieux atlas (ρ = −0.050, SE_ρ_ = 0.011, *p*_FDR_ **= 4.47 × 10^−3^, *p_raw _* = 1.45 × 10^−5^), thickness of the left medial occipitotemporal sulcus and lingual sulcus in the Destrieux atlas (ρ = −0.047, SE_ρ_ = 0.012, *p*_FDR_ **= 9.19 × 10^−3^, *p_raw _* = 5.00 × 10^−5^), and thickness of the left superior occipital gyrus in the Destrieux atlas (ρ = −0.047, SE_ρ_ = 0.012, *p*_FDR_ **= 9.19 × 10^−3^, *p_raw _* = 6.78 × 10^−5^). Sensitivity analyses identified the ventral posterior cingulate gyrus in impaired individuals, insula in unimpaired individuals, and the pars orbitalis and thalamic nuclei in both.

Figure 2B illustrates surface area measures from the Destrieux atlas colored by genetic covariance with memslopes in all, impaired, and unimpaired individuals, including the *APOE* region. Highlighted regions include the cingulate cortex and precuneus. The total number of significant structural IDPs for memslopes grouped by brain region is illustrated in Figure 2C (right), with highest counts in frontal and parietal regions as well as projection tracts.

### 3.3 Genetic covariance between functional IDPs and memory

**FIGURE 4:**
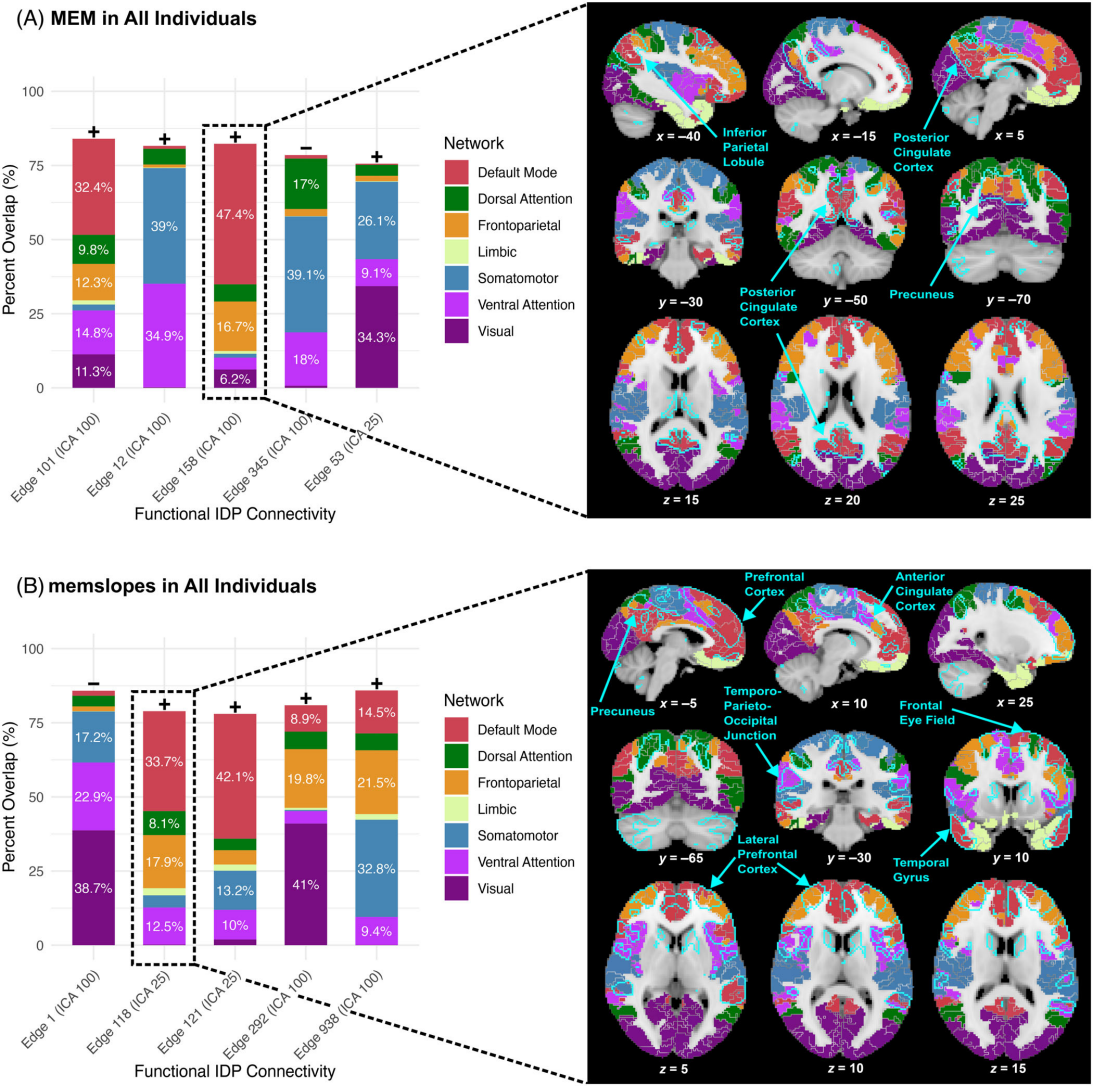
Percent overlap of top functional IDPs with Yeo networks. Left: Bar charts show percent of overlapping volume between functional IDPs and each of the seven Yeo networks (Default Mode, Dorsal Attention, Frontoparietal, Limbic, Somatomotor, Ventral Attention, Visual) for MEM (A) and memslopes (B). Plus and minus signs indicate positive and negative genetic covariance with memory, respectively. Notably, edges 158 and 101 of 100‐dimensional ICA (MEM) as well as edges 118 and 121 of 25‐dimensional ICA (memslopes) showed large overlaps with the Default Mode Network. On the right, anatomical illustrations of edges 158 and 118 highlight regions such as the posterior cingulate cortex, inferior parietal lobule, precuneus, prefrontal cortex, and anterior cingulate cortex.

The top functional IDPs that exhibited greatest genetic covariance with memory in all, impaired, and unimpaired individuals, including the *APOE* region, are shown in Figure 1 (bottom panel) for MEM and Figure 3 (bottom panel) for memslopes. For the main MEM analysis, top functional IDPs included rfMRI connectivity of edge 158 (ρ = 0.039, SE_ρ_ = 7.96 × 10^−3^, *p*_FDR_ **= 2.17 × 10^−3^, *p_raw _* = 8.50 × 10^−7^), edge 101 (ρ = 0.044, SE_ρ_ = 9.33 × 10^−3^, *p*_FDR_ **= 2.40 × 10^−3^, *p_raw _* = 2.59 × 10^−6^), edge 12 (ρ = 0.037, SE_ρ_ = 8.79 × 10^−3^, *p*_FDR_ **= 7.52 × 10^−3^, *p_raw _* = 2.27 × 10^−5^), and edge 345 (ρ = −0.033, SE_ρ_ = 7.97 × 10^−3^, *p*_FDR_ **= 9.88 × 10^−3^, *p_raw _* = 3.73 × 10^−5^) in the 100‐dimensional ICA, as well as rfMRI connectivity of edge 53 (ρ = 0.037, SE_ρ_ = 8.35 × 10^−3^, *p*_FDR_ **= 5.21 × 10^−3^, *p_raw _* = 7.49 × 10^−6^) in the 25‐dimensional ICA. As shown in Figure 4A (left), edges 158 and 101 of the 100‐dimensional ICA show large overlaps with the DMN (47.4%, 32.4%, respectively; average overlap 21.4% among ICA100 edges), which was also observed in analyses with impaired and unimpaired individuals (Table S16). Figure 4A (right) provides an anatomical illustration of edge 158, highlighting regions such as the posterior cingulate cortex, the inferior parietal lobule, and the precuneus. For functional network overlap of each edge, see Table S3. Further visualizations of these edges can be found in the UKB edge browsers (https://www.fmrib.ox.ac.uk/ukbiobank/).

For the main memslopes analysis, top functional IDPs included rfMRI connectivity of edge 1 (ρ = −0.052, SE_ρ_ = 9.12 × 10^−3^, *p*_FDR_ **= 3.23 × 10^−5^, *p_raw _* = 1.16 × 10^−8^), edge 292 (ρ = 0.040, SE_ρ_ = 8.14 × 10^−3^, *p*_FDR_ **= 7.77 × 10^−4^, *p_raw _* = 8.38 × 10^−7^), and edge 938 (ρ = 0.038, SE_ρ_ = 8.39 × 10^−3^, *p*_FDR_ **= 3.76 × 10^−3^, *p_raw _* = 8.11 × 10^−6^) in the 100‐dimensional ICA, as well as edge 118 (ρ = 0.038, SE_ρ_ = 7.61 × 10^−3^, *p*_FDR_ **= 7.77 × 10^−4^, *p_raw _* = 6.63 × 10^−7^) and edge 121 (ρ = 0.035, SE_ρ_ = 7.72 × 10^−3^, *p*_FDR_ **= 3.76 × 10^−3^, *p_raw _* = 7.36 × 10^−6^) in the 25‐dimensional ICA. As shown in Figure 4B (left), edges 118 and 121 of the 25‐dimensional ICA show large overlaps with the DMN (33.7%, 42.1%, respectively; average overlap 20.1% among ICA25 edges). These patterns were also observed in analyses with unimpaired individuals (Table S17). Figure 4B (right) provides an anatomical illustration of edge 118, highlighting multiple regions of the prefrontal cortex, anterior cingulate cortex, and temporal gyrus as well as temporo‐parieto‐occipital junction.

## 4 DISCUSSION

This study leveraged summary statistics results from our group's prior large‐scale GWAS on memory performance and decline^5^ as well as the 2021 UKB GWAS^8^ to examine genetic covariance between memory and IDPs. We found wide‐ranging significant IDPs of diffusion, structural, and functional modalities, with the most pronounced effects centered within the medial temporal lobe and other memory‐relevant structures, as well as regions within the DMN. These findings largely parallel AD brain vulnerability and highlight the genetic relationship between neuroimaging measures and memory.

Top structural IDPs in our cross‐sectional analysis included the lingual gyrus, fusiform gyrus, and anterior cingulate cortex, which have been shown to be functionally connected with medial temporal lobe structures such as the hippocampal nuclei and entorhinal cortex.^19^ Our results also highlighted various regions in the lateral occipital cortex, which, along with the fusiform gyrus, has implications in visual and semantic processing, memory, and recall.^20^, ^21^ In our longitudinal analysis, the top structural IDPs included the pars orbitalis, insula, posterior cingulate cortex, and the thalamus. Notably, Lin and colleagues found in a previous study that cognitively unimpaired *APOE* ε4 carriers showed higher activity in the pars orbitalis (the rostral‐most portion of the inferior frontal gyrus) and the insula compared to both *APOE* ε4 non‐carriers and *APOE* ε4 carriers with MCI status, suggesting that higher activity in these frontal regions may have a protective effect against AD genetic risk.^22^ On the other hand, the posterior cingulate cortex is a well‐established component of the DMN,^23^, ^24^, ^25^ which has many implications in cognitive deficits associated with neurological disorders and is consistent with our functional IDP findings. While the implications of the thalamus in AD diagnosis and treatment have historically been underexplored, emerging evidence suggests that it may be impacted in early stages of AD and show disrupted connections with DMN nodes, in particular the posterior cingulate cortex and inferior parietal lobule.^26^, ^27^, ^28^

Top diffusion IDPs in both our cross‐sectional and longitudinal analyses included the fornix, and the cingulum was identified as a top IDP in our longitudinal analyses, which is consistent with previous literature linking limbic white matter macro‐ and microstructure to cognitive performance in both pathological and non‐pathological aging.^29^, ^30^, ^31^, ^32^, ^33^ Specifically, the fornix is the major efferent tract from the hippocampus, and its integrity has been found to predict both hippocampal volume and memory performance.^34^, ^35^, ^36^ Microstructure of the cingulum, an association tract connecting frontal, parietal, and medial temporal lobes, has been associated with episodic^37^ and verbal memory performance^38^ in older adults without dementia. Notably, both the cingulum and the fornix were identified by our group to be sensitive to memory performance and decline^9^ in normal and abnormal aging as well as to AD clinical staging.^10^ Our current findings reiterate the involvement of these white matter tracts in memory performance and highlight the potential presence of a genetic relationship.

Top functional IDPs in both our cross‐sectional and longitudinal analyses exhibited large spatial overlaps with the DMN as defined in the Yeo atlas.^16^ The DMN is a large‐scale network characterized by high intrinsic activity at rest and deactivation during focused task performance, with demonstrated involvement in memory encoding and consolidation.^23^, ^39^, ^40^ Disruptions in the functional connectivity of this network have been consistently found to distinguish patients with MCI or AD from healthy controls.^41^, ^42^, ^43^, ^44^, ^45^ Moreover, the location of such disturbances in AD patients has been found to overlap with patterns of amyloid deposition,^46^, ^47^ and tau burden within the network has been found to predict their cognitive decline one year later.^48^ Importantly, several of the DMN regions were also identified in our structural analyses as top IDPs showing greatest genetic covariance with memory, including the cingulate cortex (overlaps with both anterior and posterior portions), precuneus, and inferior frontal gyrus.^40^, ^41^, ^49^ This convergence of functional and structural evidence suggests a genetic relationship between the integrity of the DMN and memory function.

Furthermore, most of the aforementioned IDPs (anterior cingulate gyrus, insula, and pars orbitalis in the structural analyses; fornix in the diffusion analyses; DMN in the functional analyses) were identified in both the main and cognitively unimpaired analyses. Analyses in the impaired group identified regions such as the fusiform gyrus and ventral posterior cingulate cortex. These findings are largely consistent with our regional hypotheses and support the idea that the observed genetic relationships are not driven by impaired status.

This study has several strengths, in particular the pairing of our previous large‐scale GWAS on memory performance/decline with the UKB neuroimaging phenotype GWAS to explore the genetic relationship between memory and brain traits. Additionally, our genetic covariance analyses evaluated multimodal neuroimaging measures, which allowed for a more comprehensive characterization of genetic relationships spanning diffusion, structural, and resting‐state functional phenotypes. While one potential limitation of the study is the relatively low heritability of resting‐state functional IDPs, our findings regarding the DMN's involvement in memory are strengthened by several of its structural components also being identified as showing strong genetic covariance with memory in the structural analyses (posterior cingulate cortex, cingulum bundle).^50^ Another limitation is that our cognitively unimpaired group comprised individuals without a clinical diagnosis of MCI or AD; however, it is possible that they possess abnormal neuropathology despite the absence of overt cognitive impairment, which should be considered when interpreting our findings. Furthermore, our memory GWAS primarily included highly educated, NHW individuals, which limited the generalizability of our results. Finally, while our results suggest genetic pleiotropy between late‐life memory and mid‐life brain structure/function, further work is needed to determine whether this reflects causal effects or linkage disequilibrium.

This genetic covariance study leveraged two sets of large‐scale GWAS results to investigate the genetic relationship of diffusion, structural, and functional brain‐imaging phenotypes with memory performance and decline. Top mid‐life brain traits showing shared genetic architecture with late‐life memory highlight the medial temporal lobe and related structures as well as frontal regions and regions within the DMN, largely paralleling AD brain vulnerability in both structure and function. Our findings suggest that these measures may be linked to risk pathways for AD‐related cognitive decline and serve as potential drug targets for AD therapeutic strategies.

## CONFLICT OF INTEREST STATEMENT

TJH has served on the scientific advisory board for Vivid Genomics and serves as deputy editor and senior associate editor for *Alzheimer's & Dementia: TRCI* and *Alzheimer's & Dementia* respectively. Other authors declare no conflicts of interest. Author disclosures are available in the Supporting Information.

## CONSENT STATEMENT

Consent was not necessary since this study constituted a secondary data analysis of published GWAS summary statistics.

## Supporting information



Supporting Information

Supporting Information

## Data Availability

The memory performance and decline GWAS summary statistics can be accessed on NIAGADS (https://dss.niagads.org/datasets/; accession: NG00159). The UK Biobank brain‐imaging derived phenotype GWAS summary statistics can be accessed on the Oxford Brain Imaging Genetics Server (https://open.win.ox.ac.uk/ukbiobank/big40/).

## References

[1] Breijyeh Z , Karaman R . Comprehensive review on Alzheimer's disease: causes and treatment. Molecules. 2020;25(24):24. doi:10.3390/molecules25245789 PMC7764106 33302541

[2] Alzheimer's Association . Alzheimer's association 2025 Alzheimer's disease facts and figures. Alzheimers Dement. 2025;21(4)e70235. 10.1016/j.jalz.2016.03.001 27570871

[3] Lambert JC , Ibrahim‐Verbaas CA , Harold D , et al. Meta‐analysis of 74,046 individuals identifies 11 new susceptibility loci for Alzheimer's disease. Nat Genet. 2013;45(12):1452‐1458. doi:10.1038/ng.2802 24162737 PMC3896259

[4] Kunkle BW , Grenier‐Boley B , Sims R , et al. Genetic meta‐analysis of diagnosed Alzheimer's disease identifies new risk loci and implicates Aβ, tau, immunity and lipid processing. Nat Genet. 2019;51(3):414‐430. doi:10.1038/s41588-019-0358-2 30820047 PMC6463297

[5] Archer DB , Eissman JM , Mukherjee S , et al. Longitudinal change in memory performance as a strong endophenotype for Alzheimer's disease. Alzheimers Dement. 2024;20(2):1268‐1283. doi:10.1002/alz.13508 37985223 PMC10896586

[6] Lahti J , Tuominen S , Yang Q , et al. Genome‐wide meta‐analyses reveal novel loci for verbal short‐term memory and learning. Mol Psychiatry. 2022;27(11):4419‐4431. doi:10.1038/s41380-022-01710-8 35974141 PMC9734053

[7] Elliott LT , Sharp K , Alfaro‐Almagro F , et al. Genome‐wide association studies of brain imaging phenotypes in UK Biobank. Nature. 2018;562(7726):210‐216. doi:10.1038/s41586-018-0571-7 30305740 PMC6786974

[8] Smith SM , Douaud G , Chen W , et al. An expanded set of genome‐wide association studies of brain imaging phenotypes in UK Biobank. Nat Neurosci. 2021;24(5):737‐745. doi:10.1038/s41593-021-00826-4 33875891 PMC7610742

[9] Peter C , Sathe A , Shashikumar N , et al. White matter abnormalities and cognition in aging and Alzheimer disease. JAMA Neurol. 2025;82(8):825‐836. doi:10.1001/jamaneurol.2025.1601 40513084 PMC12150229

[10] Yang Y , Schilling K , Shashikumar N , et al. White matter microstructural metrics are sensitively associated with clinical staging in Alzheimer's disease. Alzheimers Dement Diagn Assess Dis Monit. 2023;15(2):e12425. doi:10.1002/dad2.12425 PMC10192723 37213219

[11] Mukherjee S , Choi SE , Lee ML , et al. Cognitive domain harmonization and cocalibration in studies of older adults. Neuropsychology. 2023;37(4):409‐423. doi:10.1037/neu0000835 35925737 PMC9898463

[12] Alfaro‐Almagro F , Jenkinson M , Bangerter NK , et al. Image processing and quality control for the first 10,000 brain imaging datasets from UK Biobank. NeuroImage. 2018;166:400‐424. doi:10.1016/j.neuroimage.2017.10.034 29079522 PMC5770339

[13] Zhang Y , Brady M , Smith S . Segmentation of brain MR images through a hidden Markov random field model and the expectation‐maximization algorithm. IEEE Trans Med Imaging. 2001;20(1):45‐57. doi:10.1109/42.906424 11293691

[14] Patenaude B , Smith SM , Kennedy DN , Jenkinson M . A Bayesian model of shape and appearance for subcortical brain segmentation. NeuroImage. 2011;56(3):907‐922. doi:10.1016/j.neuroimage.2011.02.046 21352927 PMC3417233

[15] Schaefer A , Kong R , Gordon EM , et al. Local‐global parcellation of the human cerebral cortex from intrinsic functional connectivity MRI. Cereb Cortex. 2018;28(9):3095‐3114. doi:10.1093/cercor/bhx179 28981612 PMC6095216

[16] Thomas Yeo BT , Krienen FM , Sepulcre J , et al. The organization of the human cerebral cortex estimated by intrinsic functional connectivity. J Neurophysiol. 2011;106(3):1125‐1165. doi:10.1152/jn.00338.2011 21653723 PMC3174820

[17] Lu Q , Li B , Ou D , et al. A powerful approach to estimating annotation‐stratified genetic covariance via GWAS summary statistics. Am J Hum Genet. 2017;101(6):939‐964. doi:10.1016/j.ajhg.2017.11.001 29220677 PMC5812911

[18] Benjamini Y , Hochberg Y . Controlling the False Discovery rate: a practical and powerful approach to multiple testing. J R Stat Soc Ser B Methodol. 1995;57(1):289‐300. doi:10.1111/j.2517-6161.1995.tb02031.x

[19] Smagula SF , Karim HT , Rangarajan A , et al. Association of hippocampal substructure resting‐state functional connectivity with memory performance in older adults. Am J Geriatr Psychiatry. 2018;26(6):690‐699. doi:10.1016/j.jagp.2018.03.003 29628321 PMC5993618

[20] Simons JS , Koutstaal W , Prince S , Wagner AD , Schacter DL . Neural mechanisms of visual object priming: evidence for perceptual and semantic distinctions in fusiform cortex. NeuroImage. 2003;19(3):613‐626. doi:10.1016/S1053-8119(03)00096-X 12880792

[21] Davis SW , Geib BR , Wing EA , et al. Visual and semantic representations predict subsequent memory in perceptual and conceptual memory tests. Cereb Cortex. 2021;31(2):974‐992. doi:10.1093/cercor/bhaa269 32935833 PMC8485078

[22] Lin F , Ren P , Lo RY , et al. Insula and inferior frontal gyrus’ activities protect memory performance against Alzheimer's pathology in old age. J Alzheimers Dis JAD. 2017;55(2):669‐678. doi:10.3233/JAD-160715 27716674 PMC5531269

[23] Raichle ME . The brain's default mode network. Annu Rev Neurosci. 2015;38(1):433‐447. doi:10.1146/annurev-neuro-071013-014030 25938726

[24] Smallwood J , Bernhardt BC , Leech R , Bzdok D , Jefferies E , Margulies DS . The default mode network in cognition: a topographical perspective. Nat Rev Neurosci. 2021;22(8):503‐513. doi:10.1038/s41583-021-00474-4 34226715

[25] Menon V . 20 years of the default mode network: a review and synthesis. Neuron. 2023;111(16):2469‐2487. doi:10.1016/j.neuron.2023.04.023 37167968 PMC10524518

[26] Alderson T , Kehoe E , Maguire L , et al. Disrupted thalamus white matter anatomy and posterior default mode network effective connectivity in amnestic mild cognitive impairment. Front Aging Neurosci. 2017;9:370. doi:10.3389/fnagi.2017.00370 29167639 PMC5682321

[27] Aggleton JP , Pralus A , Nelson AJD , Hornberger M . Thalamic pathology and memory loss in early Alzheimer's disease: moving the focus from the medial temporal lobe to Papez circuit. Brain. 2016;139(7):1877‐1890. doi:10.1093/brain/aww083 27190025 PMC4939698

[28] Biesbroek JM , Verhagen MG , van der Stigchel S , Biessels GJ . When the central integrator disintegrates: a review of the role of the thalamus in cognition and dementia. Alzheimers Dement. 2023;20(3):2209‐2222. doi:10.1002/alz.13563 38041861 PMC10984498

[29] Hirschfeld LR , Deardorff R , Chumin EJ , et al. White matter integrity is associated with cognition and amyloid burden in older adult Koreans along the Alzheimer's disease continuum. Alzheimers Res Ther. 2023;15(1):218. doi:10.1186/s13195-023-01369-5 38102714 PMC10725037

[30] Liu ZY , Zhai FF , Han F , et al. Regional disruption of white matter integrity and network connectivity are related to cognition. J Alzheimer's Dis. 2022;89(2):593‐603. doi:10.3233/JAD-220191 35912739

[31] Groechel RC , Alosco ML , Dixon D , et al. Associations between white matter integrity of the cingulum bundle, surrounding gray matter regions, and cognition across the dementia continuum. J Comp Neurol. 2023;531(18):2162‐2171. doi:10.1002/cne.25564 38010204 PMC10841586

[32] Chen Y , Wang Y , Song Z , Fan Y , Gao T , Tang X . Abnormal white matter changes in Alzheimer's disease based on diffusion tensor imaging: a systematic review. Ageing Res Rev. 2023;87:101911. doi:10.1016/j.arr.2023.101911 36931328

[33] Operto G , Cacciaglia R , Grau‐Rivera O , et al. White matter microstructure is altered in cognitively normal middle‐aged *APOE*‐ε4 homozygotes. Alzheimers Res Ther. 2018;10(1):48. doi:10.1186/s13195-018-0375-x 29793545 PMC5968505

[34] Gazes Y , Li P , Sun E , Razlighi Q , Tsapanou A . Age specificity in fornix‐to‐hippocampus association. Brain Imaging Behav. 2019;13(5):1444‐1452. doi:10.1007/s11682-018-9958-1 30187206 PMC6401355

[35] Douet V , Chang L . Fornix as an imaging marker for episodic memory deficits in healthy aging and in various neurological disorders. Front Aging Neurosci. 2015;6:343. doi:10.3389/fnagi.2014.00343 25642186 PMC4294158

[36] Fletcher E , Raman M , Huebner P , et al. Loss of fornix white matter volume as a predictor of cognitive impairment in cognitively normal elderly individuals. JAMA Neurol. 2013;70(11):1389‐1395. doi:10.1001/jamaneurol.2013.3263 24018960 PMC4059679

[37] Ezzati A , Katz MJ , Lipton ML , Zimmerman ME , Lipton RB . Hippocampal volume and cingulum bundle fractional anisotropy are independently associated with verbal memory in older adults. Brain Imaging Behav. 2016;10(3):652‐659. doi:10.1007/s11682-015-9452-y 26424564 PMC4816657

[38] van der Holst HM , Tuladhar AM , van Norden AGW , et al. Microstructural integrity of the cingulum is related to verbal memory performance in elderly with cerebral small vessel disease: the RUN DMC study. NeuroImage. 2013;65:416‐423. doi:10.1016/j.neuroimage.2012.09.060 23032491

[39] Raichle ME , MacLeod AM , Snyder AZ , Powers WJ , Gusnard DA , Shulman GL . A default mode of brain function. Proc Natl Acad Sci. 2001;98(2):676‐682. doi:10.1073/pnas.98.2.676 11209064 PMC14647

[40] Mohan A , Roberto AJ , Mohan A , et al. The significance of the default mode network (DMN) in neurological and neuropsychiatric disorders: a review. Yale J Biol Med. 2016;89(1):49‐57. 27505016 PMC4797836

[41] Ibrahim B , Suppiah S , Ibrahim N , et al. Diagnostic power of resting‐state fMRI for detection of network connectivity in Alzheimer's disease and mild cognitive impairment: a systematic review. Hum Brain Mapp. 2021;42(9):2941‐2968. doi:10.1002/hbm.25369 33942449 PMC8127155

[42] Dennis EL , Thompson PM . Functional brain connectivity using fmri in aging and Alzheimer's disease. Neuropsychol Rev. 2014;24(1):49‐62. doi:10.1007/s11065-014-9249-6 24562737 PMC4109887

[43] Gili T , Cercignani M , Serra L , et al. Regional brain atrophy and functional disconnection across Alzheimer's disease evolution. J Neurol Neurosurg Psychiatry. 2011;82(1):58‐66. doi:10.1136/jnnp.2009.199935 20639384

[44] Grieder M , Wang DJJ , Dierks T , Wahlund LO , Jann K . Default mode network complexity and cognitive decline in mild Alzheimer's disease. Front Neurosci. 2018;12:770. doi:10.3389/fnins.2018.00770 30405347 PMC6206840

[45] Hsu YH , Huang SM , Lin SY , Yang JJ , Tu MC , Kuo LW . Prospective memory and default mode network functional connectivity in normal and pathological aging. J Alzheimer's Dis. 2022;86(2):753‐762. doi:10.3233/JAD-215293 35124645

[46] Hedden T , Dijk KRAV , Becker JA , et al. Disruption of functional connectivity in clinically normal older adults harboring amyloid burden. J Neurosci. 2009;29(40):12686‐12694. doi:10.1523/JNEUROSCI.3189-09.2009 19812343 PMC2808119

[47] Mormino EC , Smiljic A , Hayenga AO , et al. Relationships between beta‐amyloid and functional connectivity in different components of the default mode network in aging. Cereb Cortex. 2011;21(10):2399‐2407. doi:10.1093/cercor/bhr025 21383234 PMC3169663

[48] Katsumi Y , Howe IA , Eckbo R , et al. Default mode network tau predicts future clinical decline in atypical early Alzheimer's disease. Brain J Neurol. 2025;148(4):1329‐1344. doi:10.1093/brain/awae327 PMC11969453 39412999

[49] Uddin LQ , Clare Kelly AM , Biswal BB , Xavier Castellanos F , Milham MP . Functional connectivity of default mode network components: correlation, anticorrelation, and causality. Hum Brain Mapp. 2008;30(2):625‐637. doi:10.1002/hbm.20531 PMC3654104 18219617

[50] Teipel SJ , Bokde ALW , Meindl T , et al. White matter microstructure underlying default mode network connectivity in the human brain. NeuroImage. 2010;49(3):2021‐2032. doi:10.1016/j.neuroimage.2009.10.067 19878723

